# Supplementary material for: MEGH: A parametric class of general hazard models for clustered survival data
Source: Stat Methods Med Res. 2022 Jun 6;31(8):1603–16. doi: 10.1177/09622802221102620 (PMC9315191; doi:10.1177/09622802221102620)
Supplement: sj-pdf-1-smm-10.1177_09622802221102620 - Supplemental material for MEGH: A parametric class of general hazard models for clustered survival data [file sj-pdf-1-smm-10.1177_09622802221102620.pdf]

Supplementary material to "MEGH: A parametric class of general hazard models for clustered survival data"

Francisco Javier Rubio[1] **and Reza Drikvandi**[2]

[1] Department of Statistical Science, University College London, London, UK

[2] Department of Mathematical Sciences, Durham University, Durham, UK

Email: f.j.rubio@ucl.ac.uk

Email: reza.drikvandi@durham.ac.uk

1. Proof of Theorem 1

Let us first introduce some notation and preliminary results. Recall that $\boldsymbol{\eta} = (\boldsymbol{\beta}, \boldsymbol{\alpha}, \boldsymbol{\theta}, \boldsymbol{\xi})$. Let $\boldsymbol{\eta}^\star = (\boldsymbol{\beta}^\star, \boldsymbol{\alpha}^\star, \boldsymbol{\theta}^\star, \boldsymbol{\xi}^\star)$ be the true value of the parameters, which is assumed to be an interior point of the parameter space Γ, and $\dim(\Gamma) = l = l_1 + l_2$, where l_1 is the number of parameters in the regression model, $(\boldsymbol{\beta}, \boldsymbol{\alpha}, \boldsymbol{\theta})$, and l_2 is the number of parameters in the random effects distribution, $\boldsymbol{\xi}$.

Note first that, by the weak law of large numbers

$$\frac{1}{n} \log m(\boldsymbol{\eta}) \overset{P}{\to} M(\boldsymbol{\eta}),$$

as $r \to \infty$, where the function

$$\begin{aligned} M(\boldsymbol{\eta}) &= E\left[\log m_1(\boldsymbol{\eta})\right] \\ &= E_\Psi\left[\log \int \exp\left\{\ell_1(\boldsymbol{\eta}, u_1, \tilde{u}_1)\right\} dG(u_1, \tilde{u}_1)\right] \\ &= E_\Psi\left[\log \int \prod_{j=1}^{n_1} h(t_{1j} \mid \mathbf{x}_{i1}, u_1, \tilde{u}_1)^{d_{1j}} \exp\left\{-\sum_{j=1}^{n_1} H(t_{1j} \mid \mathbf{x}_{1j}, u_1, \tilde{u}_1)\right\} dG(u_1, \tilde{u}_1)\right], \end{aligned}$$

represents the expected marginal likelihood, and the expectation is taken with respect to the true generating distribution of $\mathbf{z}_1 = (\mathbf{t}_1, n_1, d_{11}, \dots, d_{1n_1}, \mathbf{x}_1)$, Ψ.

We make the following technical assumptions.

A1. The parameter space Γ is compact.

A2. Censoring is non-informative and $P(C_{ij} \geq t) > 0$, for all $t \in [0, \tau]$, where $\tau > 0$ is a constant (for instance, the end of follow-up); $j = 1, \dots, n_i$ and $i = 1, \dots, r$.

A3. (Identifiability and continuity) The baseline hazard function $h_0(t;\boldsymbol{\theta})$ is continuous for each $\boldsymbol{\theta}$ and $t > 0$, and satisfies that $h_0(\cdot;\boldsymbol{\theta}^\star)$ is different from the Weibull hazard function.

A4.

$$E_\Psi \left[\sup_{\boldsymbol{\eta}\in\Gamma} || \log m(\boldsymbol{\eta}) || \right] < \infty.$$

A5. Let $\mathcal{B}$ be an open neighbourhood around $\boldsymbol{\eta}^\star$, and suppose that

$$\int \sup_{\boldsymbol{\eta}\in\mathcal{B}} ||\nabla_{\boldsymbol{\eta}} \log m(\boldsymbol{\eta})|| d\mathbf{x} < \infty,$$

and

$$\int \sup_{\boldsymbol{\eta}\in\mathcal{B}} ||\nabla^2_{\boldsymbol{\eta},\boldsymbol{\eta}} \log m(\boldsymbol{\eta})|| d\mathbf{x} < \infty.$$

A6. The expectation matrix $\mathbf{I}(\boldsymbol{\eta}) = \text{cov}_\Psi \left[\nabla_{\boldsymbol{\eta}} \log m_1(\boldsymbol{\eta}) \right]$ exists and is positive-definite for $\boldsymbol{\eta} \in \mathcal{B}$.

A7. There exist functions $\boldsymbol{\Lambda}_{k_1 k_2 k_3}(\mathbf{z})$, such that for all $1 \leq k_1, k_2, k_3 \leq l$ and $\boldsymbol{\eta} \in \mathcal{B}$

$$\left| \frac{\partial^3}{\partial \boldsymbol{\eta}_{k_1} \partial \boldsymbol{\eta}_{k_2} \partial \boldsymbol{\eta}_{k_3}} \log m(\boldsymbol{\eta}) \right| \leq \boldsymbol{\Lambda}_{k_1 k_2 k_3}(\mathbf{z}),$$

where $E_\Psi[\boldsymbol{\Lambda}_{k_1 k_2 k_3}(\mathbf{Z})] < \infty$.

The proof is based on adapting the proof of Theorem 2.1 and Lemma 2 in [1] together with Theorem 1 from [2].

(i) By Lemma 2 in [1] together with conditions A1–A4 , the expected marginal likelihood function $M(\boldsymbol{\eta})$ is maximised at $\boldsymbol{\eta}^\star$. Then, by Theorem 2.1 in [1] and Theorem 1 from [2], it follows that the marginal maximum likelihood estimator $\widehat{\boldsymbol{\eta}} \xrightarrow{P} \boldsymbol{\eta}^\star$ as $r \to \infty$.

(ii) A second order Taylor series expansion around $\boldsymbol{\eta}^\star$ of this estimating equation leads to

$$\nabla_{\boldsymbol{\eta}} \log m(\boldsymbol{\eta}^\star) + \nabla^2_{\boldsymbol{\eta}} \log m(\boldsymbol{\eta}^\star)(\widehat{\boldsymbol{\eta}} - \boldsymbol{\eta}^\star) + \mathcal{R},$$

where $\mathcal{R}$ represents the remainder term. Following the proof of Theorem 2.1 in [1] and Theorem 1 in [2], this Taylor series expansion can be shown to have stochastically bounded residual term by using

A7. Re-arranging the first terms and multiplying by $\sqrt{r}$ together with assumptions A1-A7 and the consistency result in the previous point, it can be shown that [1, 2]

$$\sqrt{r}\,(\widehat{\boldsymbol{\eta}} - \boldsymbol{\eta}^{\star}) \overset{d}{\to} N(\mathbf{0}, \mathbf{I}(\boldsymbol{\eta}^{\star})^{-1}),$$

as $r \to \infty$.

2. Two Baseline Hazard Distributions

2.1. Power Generalised Weibull

The PGW pdf, survival function and hazard functions of the PGW are as follows [3]:

$$\begin{aligned}
f(t;\eta,\nu,\delta) &= \frac{\nu}{\delta\eta^{\nu}} t^{\nu-1}\left[1+\left(\frac{t}{\eta}\right)^{\nu}\right]^{\left(\frac{1}{\delta}-1\right)} \exp\left\{1-\left[1+\left(\frac{t}{\eta}\right)^{\nu}\right]^{\frac{1}{\delta}}\right\},\\
S(t;\eta,\nu,\delta) &= \exp\left\{1-\left[1+\left(\frac{t}{\eta}\right)^{\nu}\right]^{\frac{1}{\delta}}\right\},\\
h(t;\eta,\nu,\delta) &= \frac{\nu}{\delta\eta^{\nu}} t^{\nu-1}\left[1+\left(\frac{t}{\eta}\right)^{\nu}\right]^{\left(\frac{1}{\delta}-1\right)},
\end{aligned}$$

where $\eta > 0$ is a scale parameter and $\nu, \delta > 0$ are shape parameters.

2.2. Log-logistc

The log-logistic pdf and cdf are given by

$$f(t;\mu,\tau) = \frac{g\left(\dfrac{\log(t)-\mu}{\tau}\right)}{t\tau},$$
$$F(t;\mu,\tau) = G\left(\frac{\log(t)-\mu}{\tau}\right),$$

where $\tau > 0$, $\mu \in \mathbb{R}$, $g(t) = \dfrac{e^{-t}}{(1+e^{-t})^2}$, and $G(t) = \dfrac{1}{1+e^{-t}}$. The hazard and survival functions can be obtained as usual, $h(t;\mu,\tau) = \dfrac{f(t;\mu,\tau)}{S(t;\mu,\tau)}$ and $S(t;\mu,\tau) = 1 - F(t;\mu,\tau)$.

3. Additional Simulation Results

In this section, we report some additional results regarding the simulations for the MEGH model (9). We first present simulation results for the case when the variance of random effects is smaller, where we consider $\sigma_u = 0.5$. The results, which are shown in Figure 1, indicate that the MEGH model with the correct mixed hazard structure produces the smallest bias in this case as well.

We then present simulation results for the case when the random-effects distribution is misspecified in the simulations. For this, we generate the random effects from a two-piece normal distribution so that $\sigma_u = 1$, while we fit the model assuming the standard normal distribution for random effects. The results presented in Figure 2 suggest that the estimates from the MEGH model with the correct mixed hazard structure are quite robust with respect to the misspecification of random-effects distribution. This finding is in line with the existing literature on this type of misspecification. However, we observe that the MEGH model with both the incorrect mixed hazard structure and the misspecified random-effects distribution produces substantially inaccurate estimates.

We also present simulation results for the case when both mixed structures I and II are misspecified. For this, we generate simulated data from model (9) with the general mixed structure (1) and PGW baseline hazard. In this case, the two sets of random effects u_i and $\tilde{u}_i$ are generated from normal distributions with $\sigma_u = 1$ and $\sigma_{\tilde{u}} = 0.5$ respectively, and with $\text{cov}(\sigma_u, \sigma_{\tilde{u}}) = 0.2$. The results, which are shown in Figure 3, indicate that the estimates obtained from the MEGH-I are less affected compared to both the MEGH-II and the MLE approaches, however there is substantial bias for the estimates of the baseline hazard parameters for all methods under this misspecification.

To evaluate the behaviour of the proposed MEGH model with a different baseline hazard, we repeat the previous simulations with the log-logistic baseline hazard. From the simulation results presented in Figures 4-7, one can see that the results are pretty similar to those obtained with the PGW baseline hazard.

As asked by a referee, we also investigate the power and Type I error of the test for random effects under different number of clusters, censoring rates and variance values. For this, we calculate the rejection rate of the test for random effects across 250 simulation replications for the model with structure MEGH-I evaluated under different number of clusters $r = 12, 24$ and different censoring rates of 25% and 50%, when the random effects are generated from a normal distribution with different variance values of $\sigma_u = 0, 0.25, 0.50$.

Figure 1: The bias of the estimates from the three methods: MEGH1, MEGH2 and MLE, for all the parameters based on 250 simulation replications when the simulated data are generated from model (9) with the mixed structure I and PGW baseline hazard, and a normal distribution for the generated random effects with $\sigma_u = 0.5$.

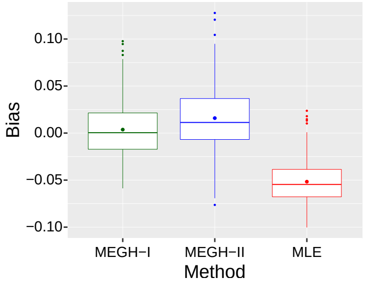

(a) Bias in estimating η

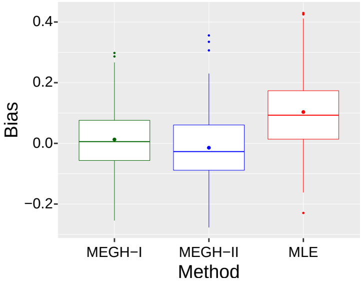

(b) Bias in estimating ν

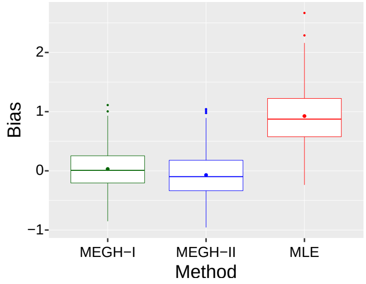

(c) Bias in estimating δ

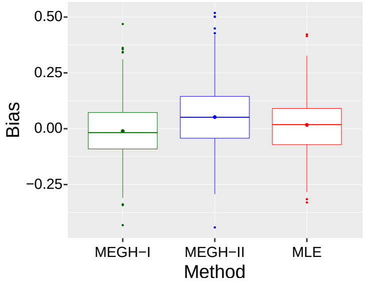

(d) Bias in estimating α

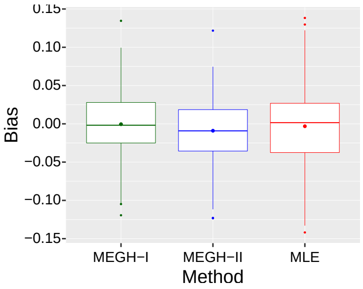

(e) Bias in estimating β_1

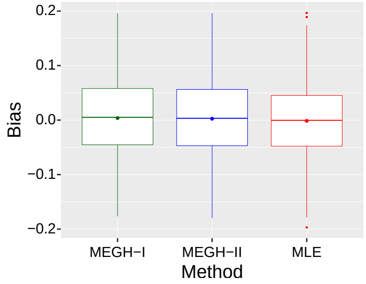

(f) Bias in estimating β_2

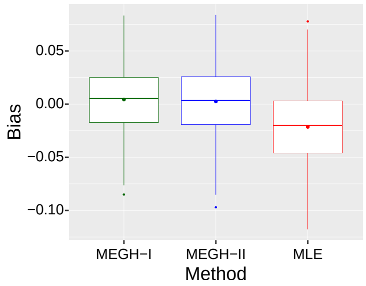

(g) Bias in estimating β_3

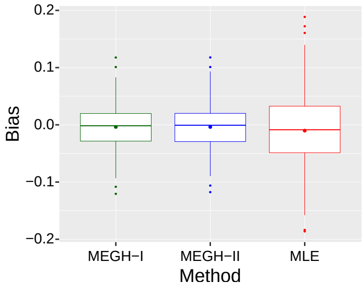

(h) Bias in estimating β_4

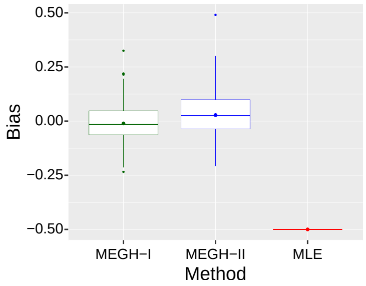

(i) Bias in estimating σ_u

The results, presented in Table 1, indicate that the Type I error of the test is at the nominal level 0.05 and the power of the test is reasonably high even with smaller number of clusters or higher censoring rate.

Finally, Figure 8 shows the confidence intervals for the leukemia data. There seem to be slight differences between the confidence intervals obtained by the MEGH model and the model ignoring random effects. One may find the parameter β_2 is not significant here using the model ignoring random effects, while the MEGH model hardly shows that. It should be pointed out that the estimate of σ_u is relatively small for this data set, and we would expect the differences to be larger if σ_u was bigger, as shown in our simulations.

Figure 2: The bias of the estimates from the three methods: MEGH1, MEGH2 and MLE, for all the parameters based on 250 simulation replications when the simulated data are generated from model (9) with the mixed structure I and PGW baseline hazard, and a two-piece normal distribution for the generated random effects with $\sigma_u = 1$.

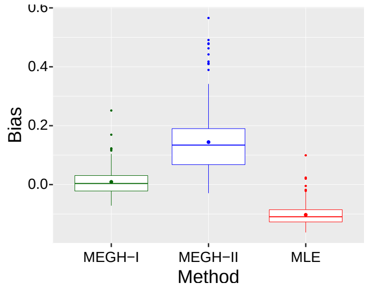

(a) Bias in estimating η

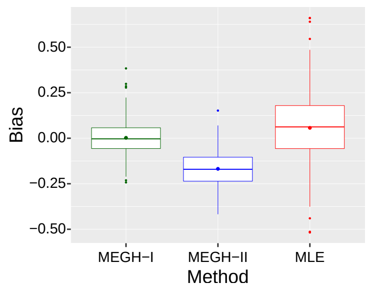

(b) Bias in estimating ν

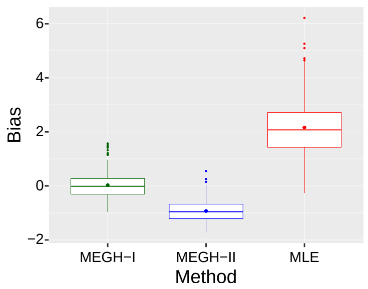

(c) Bias in estimating δ

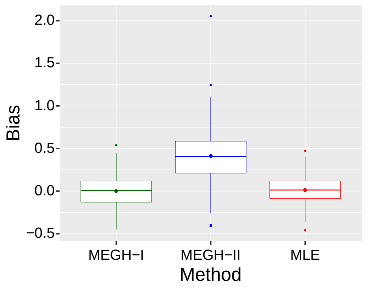

(d) Bias in estimating α

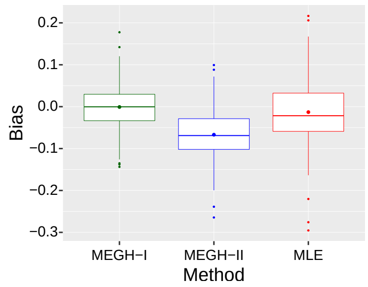

(e) Bias in estimating β_1

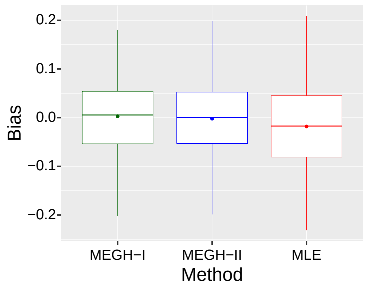

(f) Bias in estimating β_2

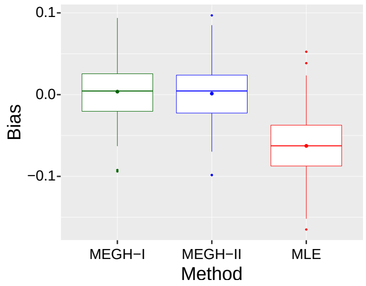

(g) Bias in estimating β_3

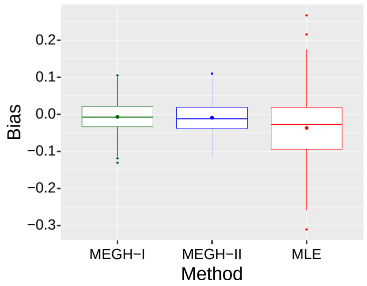

(h) Bias in estimating β_4

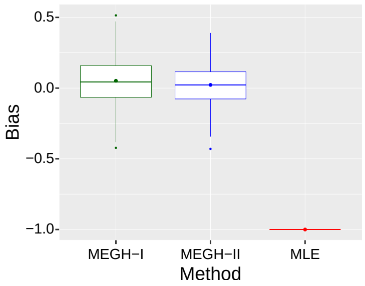

(i) Bias in estimating σ_u

Figure 3: The bias of the estimates from the three methods: MEGH1, MEGH2 and MLE, for all the parameters based on 250 simulation replications when the simulated data are generated from model (9) with the general mixed structure (1) (i.e., both mixed structures I and II are misspecified) and PGW baseline hazard. Note that the random effects u_i and $\tilde{u}_i$ are generated from normal distributions with $\sigma_u = 1$, $\sigma_{\tilde{u}} = 0.5$ and $\text{cov}(\sigma_u, \sigma_{\tilde{u}}) = 0.2$.

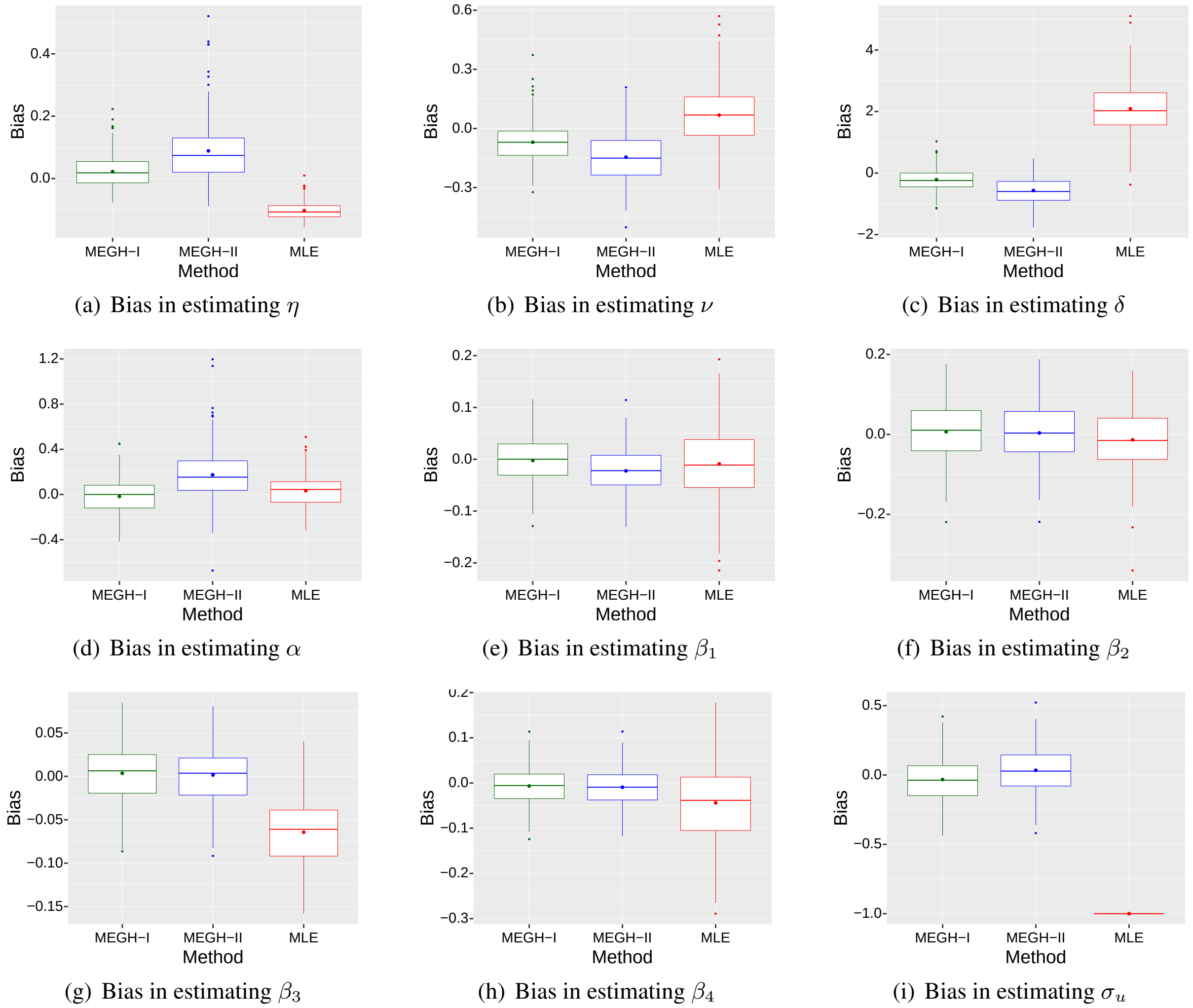

(a) Bias in estimating η (b) Bias in estimating ν (c) Bias in estimating δ

(d) Bias in estimating α (e) Bias in estimating β_1 (f) Bias in estimating β_2

(g) Bias in estimating β_3 (h) Bias in estimating β_4 (i) Bias in estimating σ_u

Figure 4: The bias of the estimates from the three methods: MEGH1, MEGH2 and MLE, for all the parameters based on 250 simulation replications when the simulated data are generated from model (9) with the mixed structure I and log-logistic baseline hazard, and a normal distribution for the generated random effects with $\sigma_u = 1$.

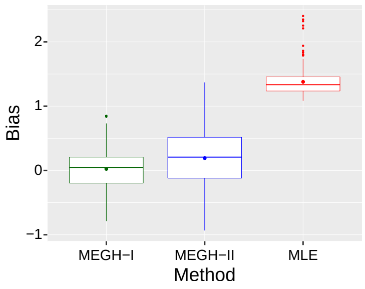

(a) Bias in estimating μ

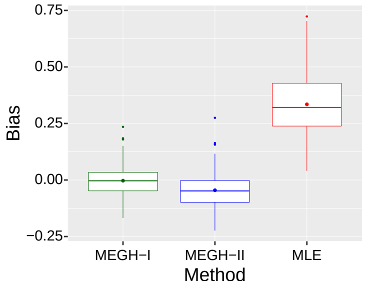

(b) Bias in estimating τ

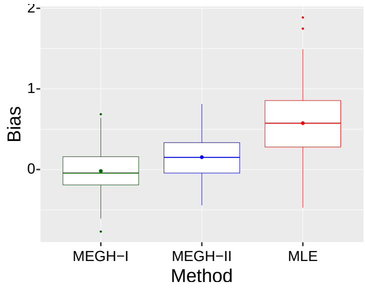

(c) Bias in estimating α

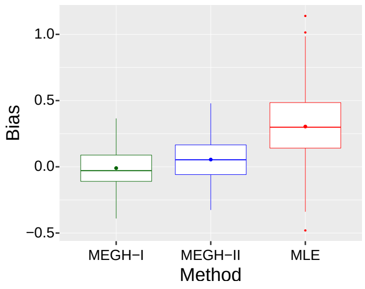

(d) Bias in estimating β_1

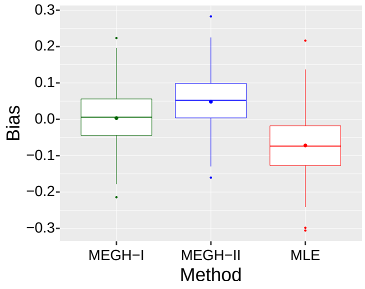

(e) Bias in estimating β_2

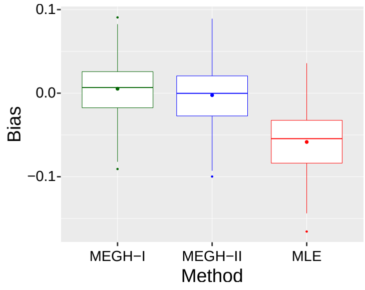

(f) Bias in estimating β_3

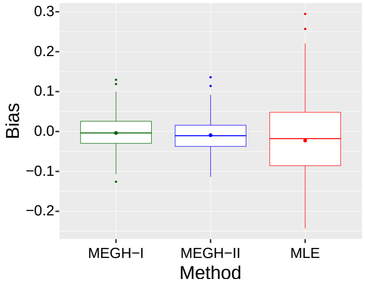

(g) Bias in estimating β_4

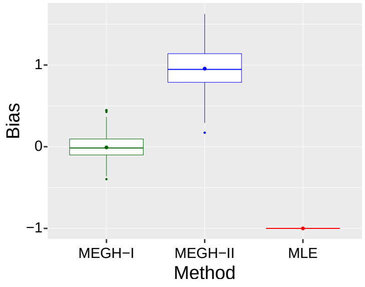

(h) Bias in estimating σ_u

Figure 5: The bias of the parameter estimates from the three methods: MEGH1, MEGH2 and MLE for all the parameters based on 250 simulation replications when the simulated data are generated from model (9) with the mixed structure II and log-logistic baseline hazard, and a normal distribution for the generated random effects with $\sigma_u = 1$.

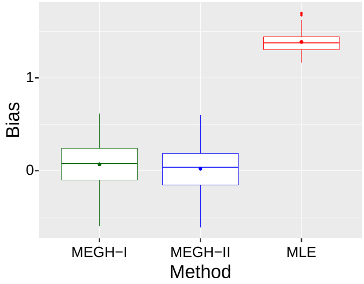

(a) Bias in estimating μ

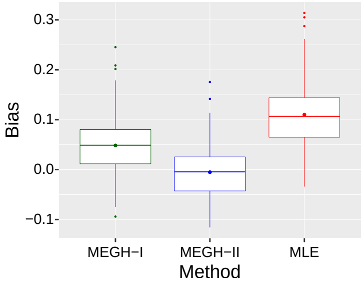

(b) Bias in estimating τ

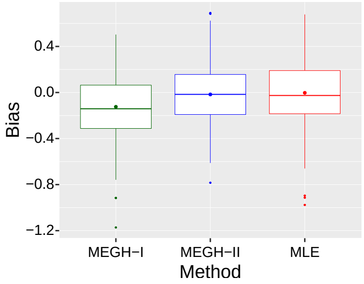

(c) Bias in estimating α

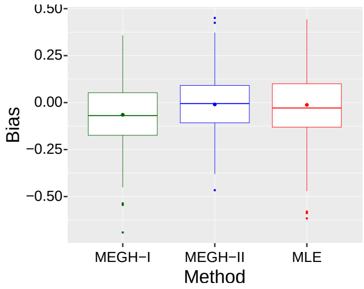

(d) Bias in estimating β_1

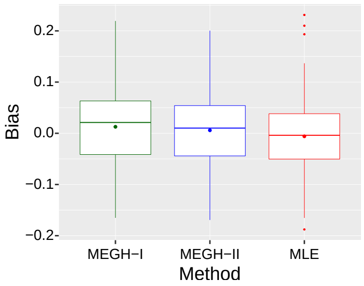

(e) Bias in estimating β_2

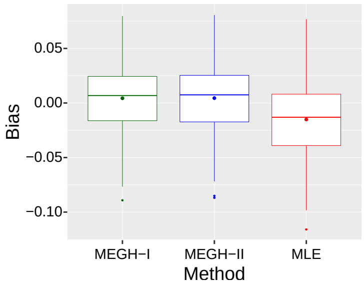

(f) Bias in estimating β_3

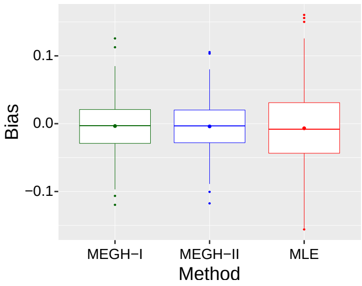

(g) Bias in estimating β_4

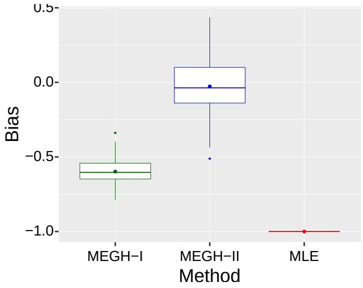

(h) Bias in estimating σ_u

Figure 6: The bias of the estimates from the three methods: MEGH1, MEGH2 and MLE, for all the parameters based on 250 simulation replications when the simulated data are generated from model (9) with the mixed structure I and log-logistic baseline hazard, and a normal distribution for the generated random effects with $\sigma_u = 0.5$.

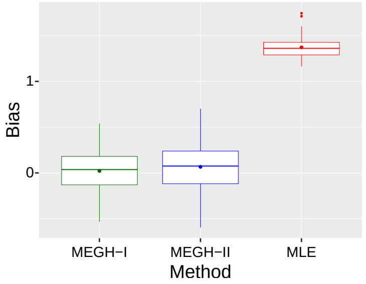

(a) Bias in estimating μ

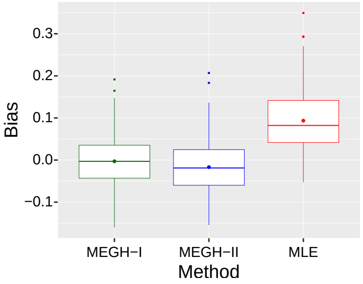

(b) Bias in estimating τ

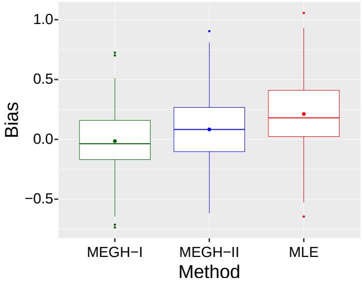

(c) Bias in estimating α

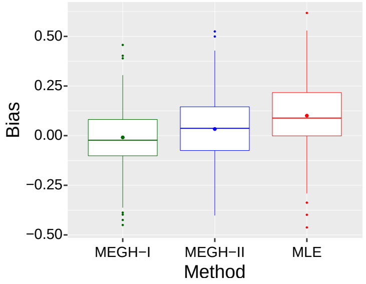

(d) Bias in estimating β_1

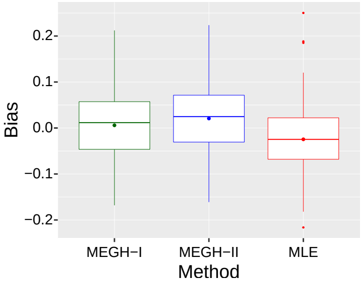

(e) Bias in estimating β_2

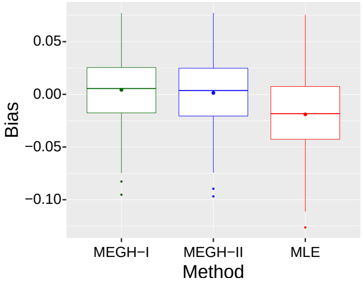

(f) Bias in estimating β_3

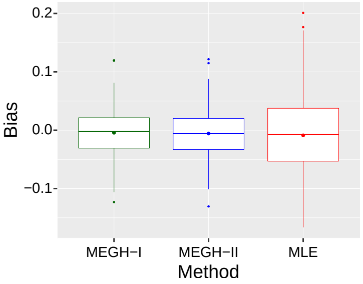

(g) Bias in estimating β_4

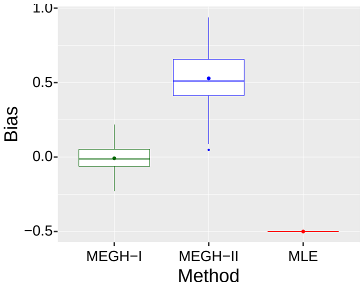

(h) Bias in estimating σ_u

Figure 7: The bias of the estimates from the three methods: MEGH1, MEGH2 and MLE, for all the parameters based on 250 simulation replications when the simulated data are generated from model (9) with the mixed structure I and log-logistic baseline hazard, and a two-piece normal distribution for the generated random effects with $\sigma_u = 1$.

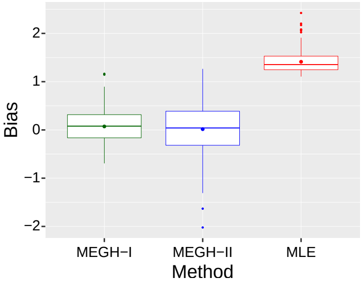

(a) Bias in estimating μ

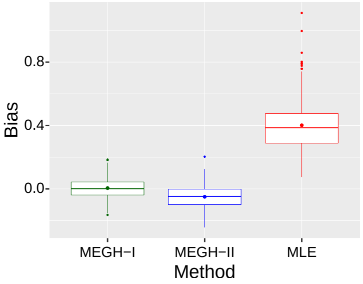

(b) Bias in estimating τ

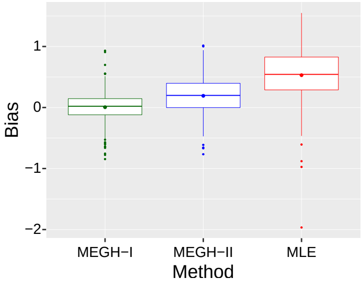

(c) Bias in estimating α

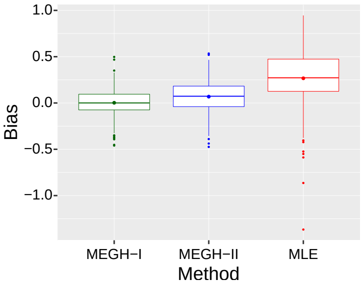

(d) Bias in estimating β_1

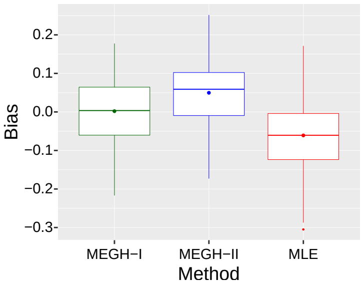

(e) Bias in estimating β_2

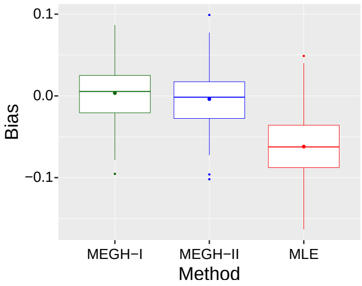

(f) Bias in estimating β_3

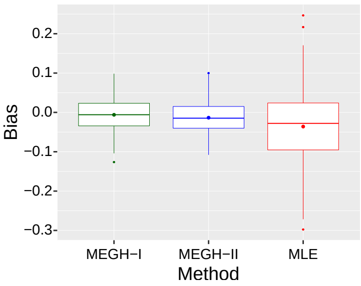

(g) Bias in estimating β_4

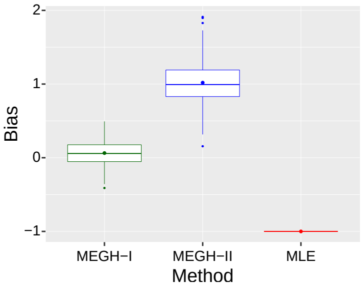

(h) Bias in estimating σ_u

Table 1: Rejection rate of the test for random effects across 250 simulation replications for the model with structure MEGH-I evaluated under different number of clusters $r = 12, 24$ and different censoring rates of 25% and 50%, when the random effects are generated from a normal distribution with different variance values of $\sigma_u = 0, 0.25, 0.50$. Note that the case $\sigma_u = 0$ is to evaluate the Type I error of the test as there is no random effect in the model, while the cases $\sigma_u = 0.25, 0.50$ are to evaluate the power of the test.

Fitted model	Number of clusters	Censoring rate	σ_u	Rejection rate
MEGH-I	12	25%	0	0.03
	12	25%	0.25	0.87
	12	25%	0.50	1.0
	12	50%	0	0.03
	12	50%	0.25	0.69
	12	50%	0.50	1.0
	24	25%	0	0.04
	24	25%	0.25	0.98
	24	25%	0.50	1.0
	24	50%	0	0.03
	24	50%	0.25	1.0
	24	50%	0.50	1.0
MEGH-II	12	25%	0	0.02
	12	25%	0.25	0.83
	12	25%	0.50	1.0
	12	50%	0	0.03
	12	50%	0.25	0.64
	12	50%	0.50	0.98
	24	25%	0	0.04
	24	25%	0.25	0.97
	24	25%	0.50	1.0
	24	50%	0	0.02
	24	50%	0.25	1.0
	24	50%	0.50	1.0

Figure 8: Leukemia data: the 90% confidence intervals for the regression parameters obtained from fitting both the MEGH-I model (solid line) and the model ignoring random effects (dashed line).

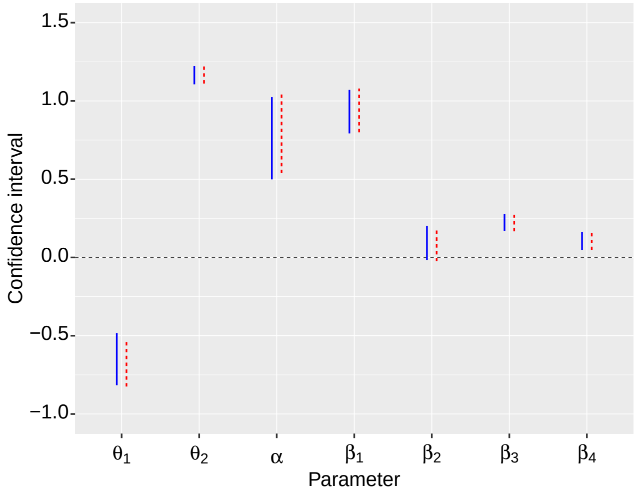

References

[1] Vatter T, Chavez-Demoulin V. Generalized additive models for conditional dependence structures. Journal of Multivariate Analysis. 2015;141:147-67.

[2] Prenen L, Braekers R, Duchateau L. Extending the Archimedean copula methodology to model multivariate survival data grouped in clusters of variable size. Journal of the Royal Statistical Society: Series B. 2017;79(2):483-505.

[3] Bagdonavicius V, Nikulin M. Accelerated life models: modeling and statistical analysis. Chapman and Hall/CRC; 2001.
